# Broiler flocks in production systems with slower-growing breeds and reduced stocking density receive fewer antibiotic treatments and have lower mortality

**DOI:** 10.1016/j.psj.2024.104197

**Published:** 2024-08-08

**Authors:** Y. Slegers, M. Hostens, M.G.R. Matthijs, J.A. Stegeman, J.J. de Wit

**Affiliations:** ⁎ Farm Animal Health, Department of Population Health Sciences, Faculty of Veterinary Medicine, Utrecht University, PO Box 80.151, 3508 TD Utrecht, The Netherlands; † Department of Animal Science, Cornell University College of Agriculture and Life Sciences, Cornell University, Ithaca, NY 14853-4801, USA; ‡ Royal GD, PO box 9, 7400 AA Deventer, The Netherlands

**Keywords:** broiler, slower-growing, antibiotic, mortality, footpad lesion

## Abstract

In the Netherlands, the number of broiler production systems with higher welfare standards, using slower-growing broilers and decreased stocking densities, has increased over the last decade. This study aimed to investigate the effect of this change on antibiotic treatments, mortality, and footpad lesions. Data from national monitoring databases from 2013 to 2021 were used, resulting in 113,380 included flocks from 917 farms. Flocks were divided into conventional (**CONV**), medium-growing (**MED**), and slow-growing (**SLOW**), based on breed and slaughter age (median age: CONV 42 d; MED 50 d; SLOW 56 d). Generalized mixed-effect models were created to compare antibiotic treatments in and after the first week, total on-farm mortality, and footpad lesion scores between these 3 production systems. Year, quarter, flock size, thinning, number of houses, and regional density of poultry farms were included as fixed effects. Random effects were farm and veterinary practice in all models, with an additional random slaughterhouse effect to describe footpad lesions. Probability of treatment in the first week of age in CONV flocks overall years (7.2%, 95% CI [5.9, 8.7]) was higher than in MED (2.0%, 95% CI [1.6, 2.5]) and SLOW flocks (1.3%, 95% CI [1.0, 1.7]). Treatment probability after the first week was similarly higher in CONV flocks (14.7%, 95% CI [12.1, 17.6]) than in MED (3.2%, 95% CI [2.5, 4.0]) and SLOW flocks (2.2%, 95% CI [1.7, 2.9]). CONV flocks had a higher mean mortality (3.2%, 95% CI [3.0, 3.4]) than MED (2.0%, 95% CI [1.9, 2.1]) and SLOW flocks (1.9%, 95% CI [1.8, 2.0]). Regarding footpad lesions, CONV flocks had the highest mean scores (range 0–200) over all years, whereas SLOW flocks had the lowest scores (CONV: 46.1, 95% CI [42.1, 50.6]; MED: 21.3, 95% CI [18.9, 24.0]; SLOW: 13.2, 95% CI [11.5, 15.1]). This analysis of data from flocks over a 9-yr period indicates that switching from conventional to alternative production systems with higher welfare standards could positively affect broiler health and antibiotic use.

## INTRODUCTION

In recent decades, the chicken meat industry has developed into a large and efficient industry. The modern conventional broiler chicken strains are characterized by their fast growth (>60 g/d), reaching a body weight of around 2.5 kg in approximately 38 d (Aviagen, 2022). However, a shift can be observed from a focus on maximizing production towards prioritizing higher welfare standards, particularly in western Europe (ECC, 2018). This leads to changes such as using slower-growing broilers and reducing stocking density.

In the Netherlands, this shift is evident in the emergence of “middle segment” broiler concepts, between conventional and organic farming, using slower-growing broiler breeds. The first major step was taken in 2008 when the Dutch Society for the Protection of Animals introduced the ‘Better Life’ system (Saatkamp et al., 2019). The requirements for broiler meat to receive one out of 3 stars include a maximum growth rate of 45 g/d, minimal slaughter age of 56 d, maximum stocking density of 25 kg/m^2^, provision of a covered outdoor area and environmental enrichments (de Jong et al., 2022). Moreover, starting in 2012, Dutch retail chains began to develop their own broiler concepts, aimed at enhancing welfare. While the exact requirements differed between these concepts, they all posed constraints on growth rate (between 45 and 50 g/d) and stocking density (between 30 and 38 kg/m^2^) (Stadig, 2019). Presently, Dutch supermarkets exclusively sell meat from slower-growing broilers, and they have pledged to sell only meat from Better Life broilers since December 2023, which means a phase-out of retail concepts (Dutch Society for the Protection of Animals, 2021). Meanwhile, conventional fast-growing broilers are still reared for other purposes such as export.

Previous studies generally agree that slower-growing broilers have improved welfare compared to conventional broilers. Many of those studies focused on leg problems and contact dermatitis. Under similar housing conditions, slower-growing chickens generally have better gait scores (Dixon, 2020; Rayner et al., 2020; Abeyesinghe et al., 2021; Baxter et al., 2021; Abdourhamane and Petek, 2022), as well as less severe footpad lesions (**FPL**) and hock burn than fast-growing chickens (Rayner et al., 2020; Abeyesinghe et al., 2021; Abdourhamane and Petek, 2022; van der Eijk et al., 2023). Studies also report lower mortality in slower-growing broilers compared to fast-growing broilers under similar commercial housing conditions (Baxter et al., 2021; Forseth et al., 2024), as well as when comparing slower-growing flocks from production systems with higher welfare standards to conventional flocks (de Jong et al., 2022). Lower mortality of slower-growing breeds is also observed in experimental settings (Fanatico et al., 2008; Dixon, 2020; Abeyesinghe et al., 2021), although other studies found no difference (Weimer et al., 2020; Torrey et al., 2020; Abdourhamane and Petek, 2022). These studies vary widely in housing, breed, and slaughter age, in addition to widely differing overall mortality, which impedes a direct comparison.

To the authors’ knowledge, no study has focused on disease incidence or antibiotic treatments of slower-growing broilers under commercial conditions. Experimental studies suggest that fast-growing chickens show an impaired immune response to infection with *Eimeria maxima* (Giles et al., 2019), and demonstrate a difference in immune response to *Salmonella* (van Hemert et al., 2006; Snyder et al., 2022; Drauch et al., 2022) compared to slower-growing chickens. Moreover, fast growth predisposes broilers to necrotic enteritis (Dierick et al., 2019). It is valuable to determine whether the suggested decrease in disease susceptibility of slower-growing chickens affects broiler health under commercial conditions. In addition, a potential effect on the use of antibiotics would be important from a public health perspective, because antibiotic use can be a driver of antibiotic resistance, one of the major public health threats of the 21st century (World Health Organization, 2021).

In this study, a comparison was made between conventional boiler flocks and slower-growing broiler flocks in alternative production systems regarding flock health. Antibiotic treatments, total mortality and footpad lesion score were analyzed. In the Netherlands, all antibiotic supplies must be registered for monitoring by the Netherlands Veterinary Medicines Authority (SDa). This, together with the ban on preventive use of antibiotics (Speksnijder et al., 2015), makes registered antibiotic treatments a suitable proxy for disease incidence, especially when combined with mortality. Mortality and FPL scores are also routinely registered. Additionally, the relationship between mortality, antibiotic treatments, and FPL was analyzed. National monitoring data spanning 9 yr (2013–2021), of which data from 2017 until 2021 included mortality and FPL scores, was used to give an as complete as possible overview of the situation in the Netherlands.

## MATERIALS AND METHODS

### Data

**Figure 1:**
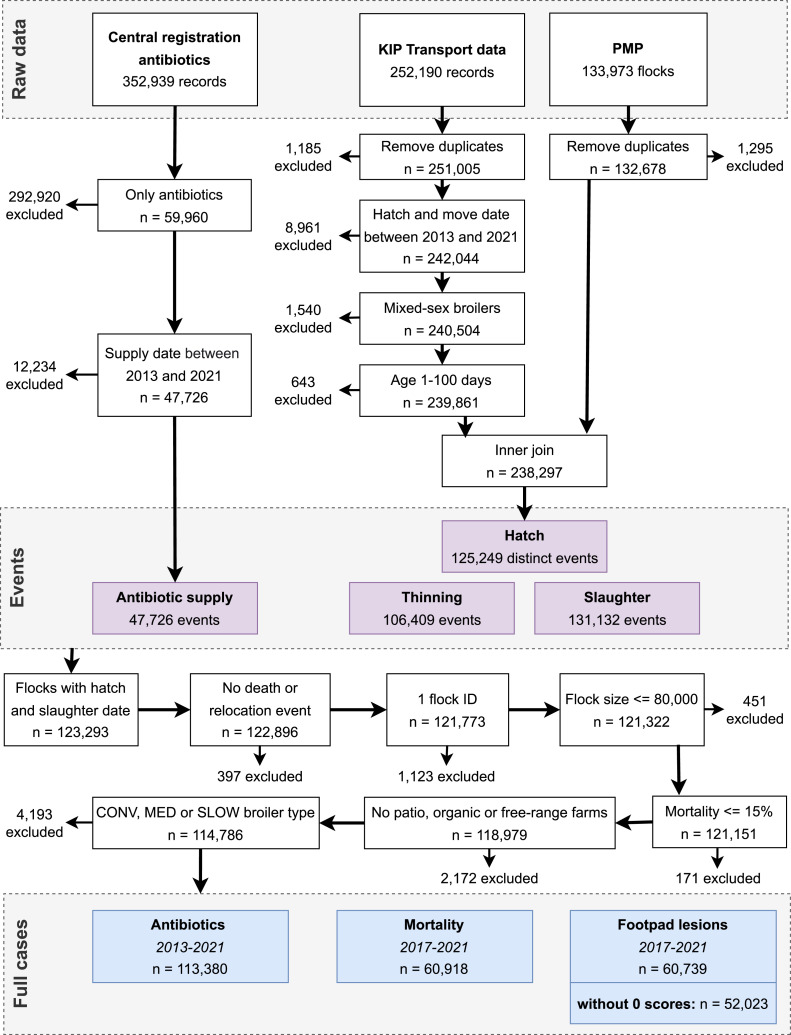
Data merge and filter steps.

This study was part of a larger EU-funded project called DECIDE (https://decideproject.eu/). In this study, national datasets were combined on flock level. A flock was defined as a group of birds placed together in a house, with a maximum of 3 d difference between hatch dates. The main databases were the *Central Registration Antibiotics* (**CRA**), in which all antibiotic supplies are registered on house level; the *Poultry Monitoring Program* (**PMP**), in which flocks are identified based on verified bird movements from the *Flock Information system Poultry* (**KIP**); and a separate extraction from the KIP database containing slaughter transport registrations, including FPL scores and mortality. The datasets included data from 2013 to 2021, but mortality and FPL scores were recorded from 2017 onwards. The PMP and KIP datasets were linked on farm (KIP number), house number, and hatch date of the flock. The resulting dataset contained information on farm and flock characteristics, and slaughter transports, including "thinning" (partial depopulation). Data from CRA was then linked to the PMP/KIP dataset on flock level using the Unique Business Number (UBN) of the farm, house number, and supply date in CRA between hatch date and slaughter date of a flock in PMP/KIP. All data merge and filter steps are shown in Figure 1.

Data cleaning started with 133,973 flocks in the PMP dataset. Only flocks with a known slaughter date were included. A flock transportation was seen as thinning if the transport date to slaughter was more than 2 d before the last slaughter date of the flock. Flocks were defined as all birds reared in 1 house at the same time; therefore, 1 flock could have multiple slaughter transports (e.g., thinning and slaughter). Flocks with a registration in KIP of death (culled due to disease) or relocation were excluded, as well as flocks with more than 1 flock identification number from PMP. Additionally, flocks with flock sizes of more than 80,000, a reported mortality of more than 15%, or an age outside the range of 1 to 100 d were excluded because these values were deemed unreliable. Furthermore, rooster or hen flocks, and farms registered as organic or free-range or using Patio systems (Vencomatic BV, Eersel, the Netherlands) in PMP were excluded because of their low numbers and distinct differences in farm management or housing conditions.

### Variables

Flocks were grouped into 3 production systems: conventional (**CONV**), medium-growing (**MED**) and slow-growing (**SLOW**). CONV flocks were from fast-growing broiler breeds (mostly Ross 308) and slaughtered at a maximum age of 46 d. MED and SLOW flocks were both from slower-growing broiler breeds, mostly Hubbard or Ross Ranger. MED flocks were slaughtered between d 46 and 55, while SLOW flocks were slaughtered at d 56 or later. An overview of the breed distribution in the 3 groups is provided in Supplementary Table S1. The use of slower-growing broilers in the Netherlands is part of broader welfare standards, either retail concepts or the Better Life system, which also restrict stocking densities. Flocks with at least 1 star of the Better Life system, with stricter welfare regulations than retail concepts, are expected to be assigned to the SLOW group based on the minimal slaughter age of 56 d, but the SLOW group is not limited to Better Life flocks. As for 4,193 flocks (3.5%), they did not fit into any of these categories and were therefore excluded.

The outcome variables of interest were antibiotic treatment, mortality, and FPL scores. Antibiotic treatment was operationalized as a binary variable indicating whether a flock was treated or not. This was divided in treatment during the first week of the production round or in the following weeks, based on the supply date of antibiotics. FPL scoring is mandatory for flocks kept at stocking densities above 39 kg/m^2^. FPL scores are routinely recorded at the slaughter line using a standardized scoring method (Rijksdienst voor Ondernemend Nederland, 2021). One hundred feet are given a score of 0, 1, or 2, corresponding to no, mild, or severe lesions, respectively. The total score is calculated with the following formula: (number of mild lesions) × 0.5 + (number of severe lesions) × 2. This results in a flock score ranging from 0 to 200. Scores from thinned batches were not included in the flock FPL score. Scores of 0 were excluded from the analysis as the distribution of scores from CONV flocks suggested that these were erroneous scores. For mortality, the total on-farm mortality at the end of the production cycle, registered by the farmer, was used. This included selective culling.

Multiple co-variates were included in this study: year and quarter of hatch and slaughter date, flock size, number of houses on the farm, whether a flock was thinned, and regional poultry farm density. Flock size, number of houses, thinning and farm density were included because they differed between CONV, MED, and SLOW flocks, and they could influence disease incidence. For example, lower poultry farm density might limit the spread of disease. Flock size was estimated with the total number of broilers transported to slaughter, including thinning. The number of houses on a farm was derived from the total number of distinct houses with registered flocks on a farm, calculated per year. This variable was divided into 4 categories: 1, 2, 3, or more than 3 houses. Poultry farm density in farms/km^2^ was calculated for each year and 2-digit postal code by dividing the number of farms in an area in a certain year, supplied by Royal GD, by the surface area of that region. Farm postal code and hatch year were used to link a flock to the correct regional poultry farm density.

### Data Analysis

All analyses were performed using R version 4.2.2 (R Core Team, 2019) through the data processing platform Databricks. The following packages were used: “lme4” (Bates et al., 2015), “glmmTMB” (Brooks et al., 2017), “dplyr” (Wickham et al., 2022), “ggplot2” (Wickham, 2009), “lsmeans” (Lenth, 2016), and “performance” (Lüdecke et al., 2021). The code scripts for all analyses can be found at https://github.com/decide-project-eu/slegers-et-al-2024.

Multiple generalized linear mixed models were created in which antibiotic treatment in the first week, antibiotic treatment after the first week, FPL scores and mortality were used as dependent variables. Generally, the first step was to fit a univariate model with production system as fixed effect. Farm and veterinary practice were included as random effects. Then, multivariable models were created with production system, year (of hatch or slaughter), quarter (of hatch or slaughter), flock size, number of houses, thinning, and farm density added as fixed effects. In all models, continuous variables were scaled and centered to aid model conversion. Model selection was performed through manual backwards stepwise selection, based on the Akaike information criterion (**AIC**). A variable was included and considered significant if it decreased the model AIC by more than 2. Multicollinearity was assessed using the variance inflation factor. Lastly, interactions with production system were added if it improved the models.

To study the relationship between production system and antibiotic treatment, both during and after the first week, logistic mixed-effects models were fitted. Year and quarter of hatch date were included. Flock size was log2-transformed in this model to better fit the observed data. The interaction between production system and hatch year was included. The relationship between mortality and antibiotic treatment was examined separately, as will be discussed later.

To assess the relationship between production system and mortality, a negative binomial model was fitted with the total dead birds as outcome variable and the log of number of birds at the start as offset. This number was calculated from the mortality and flock size at the end of the round, with the assumption that flock size was only altered by death. The interaction between production system and hatch year was included.

To assess the relationship between production system and FPL scores, a negative binomial model was fitted. Slaughterhouse was included as an additional random effect since the slaughterhouse had an effect on FPL scores in previous research (de Jong et al., 2012). Year and quarter of the slaughter date were used in this model. The interaction between production system and year, production system and flock size, and production system and thinning were included. To assess the effect that removing 0 scores had on the results, the model was also run with 0 scores included.

To explore the relationship between mortality and antibiotic treatment, firstly MED and SLOW flocks were combined into a single group. This was done because otherwise the number of flocks with mortality higher than 5% was too low. After checking for a linear relationship between mortality and log odds of antibiotic treatment, a logistic regression model for antibiotic treatment during the whole production round was fitted with mortality as fixed effect. The other fixed effects were production system, hatch year, hatch quarter, flock size (log2 transformed), thinning, and an interaction between hatch year and production system. Random effects were farm and veterinary practice. An interaction between mortality and production system was tested but inclusion of this interaction did not improve the model.

The relationship between antibiotic treatment and FPL scores was assessed using a negative binomial model with antibiotic treatment during the whole production round, production system, slaughter year, slaughter quarter, thinning and number of houses as fixed effects, as well as interactions between antibiotic treatment and production system and between year and production system.

The effect of each explanatory variable was calculated as an odds ratio (logistic regression) or rate ratio (negative binomial regression), with an accompanying Wald 95% confidence interval. A significant effect of production system, number of houses or quarter was further examined by pairwise comparisons of marginal means using Tukey's HSD test. A significant effect of hatch or slaughter year, not including interactions, was examined by pairwise comparisons using Dunnett's test with the first year (2013 or 2017) as reference group.

## RESULTS

### Descriptive Statistics

**Figure 2:**
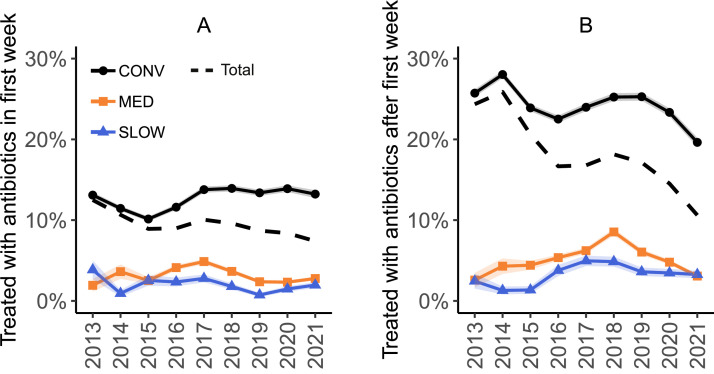
Percentage of flocks treated with antibiotics (A) in the first week, (B) after the first week of the production cycle. Colored bands indicate the standard deviation. The dashed line indicates the percentage of all flocks (CONV, MED and SLOW together). CONV: conventional; MED: medium-growing; SLOW: slow-growing.

This study included 113,380 flocks from 917 farms in the period from 2013 to 2021 (Figure 1). In 2013, only 3.1% and 2.8% of flocks were MED and SLOW, respectively (excluding organic and free-range flocks). These numbers increased every year, reaching 40.3% MED and 14.3% SLOW in 2021. Table 1 provides a summary of flock and farm characteristics. The percentage of flocks treated with antibiotics is depicted in Figure 2. Median yearly mortality and FPL scores are provided in Supplementary Figure S1 and S2, respectively. From 2017 onwards, 98.3% of CONV flocks had a registered FPL score, in contrast to 93.9% of MED and 78.2% of SLOW flocks. When 0 scores were excluded, this was 92.2%, 70.1% and 59.0% of CONV, MED and SLOW flocks, respectively.

**Table 1:** Summary statistics of conventional (**CONV**), medium slow-growing (**MED**) and slow-growing (**SLOW**) broiler flocks in the period 2013 to 2021.

		CONV	MED	SLOW
		*n*		
Farms^1^		733	645	283
Flocks	*Total*	76,301	28,400	8,679
	*2013*	9,511	311	286
	*2014*	10,592	442	540
	*2015*	10,544	1,427	592
	*2016*	9,048	3,807	768
	*2017*	8,354	4,394	1,149
	*2018*	8,249	4,382	1,235
	*2019*	7,958	4,347	1,222
	*2020*	7,175	4,963	1,357
	*2021*	4,870	4,327	1,530
Thinned		65,076 (85.3%)	415 (1.5%)	486 (5.6%)
		*Median (IQR)*		
Slaughter age (d)		42 (40–43)	50 (49–53)	56 (56–57)
Flock size		26,662 (19,306–35,134)	17,962 (12,303–24,322)	16,311 (11,135–20,718)
Number of houses on farm^2^		4 (3–6)	3 (2–5)	3 (2–4)
Poultry farm density (farms/km^2^)^2^		0.11 (0.07–0.21)	0.09 (0.05–0.19)	0.09 (0.06–0.19)

1 The sum of farms is larger than the total number of farms (n = 917) because farms can have multiple production systems.

2 Calculations are performed on flock level; farms with a greater number of houses therefore contribute proportionally more flocks, which influences the observed median.

### Antibiotic Treatment

**Figure 3:**
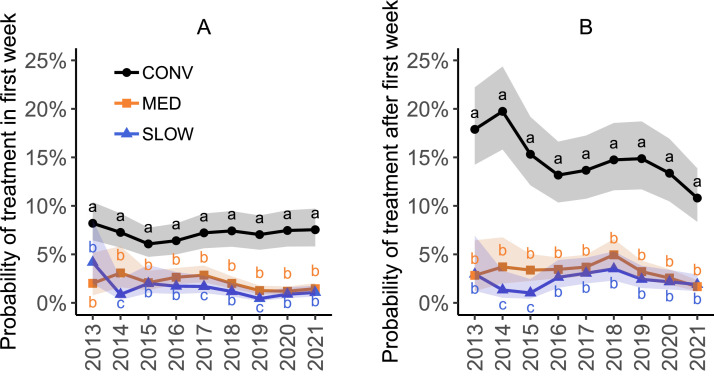
Marginal probability of treatment with antibiotics per hatch year and production system (A) in the first week, and (B) after the first week, based on the multivariate models corrected for quarter, flock size and number of houses, with random farm and veterinary practice effects. Colored bands indicate the 95% CI. Different letters (a, b, c) represent significant differences between production systems within each year. CONV: conventional; MED: medium-growing; SLOW: slow-growing.

The results of the multivariate analyses are summarized in Table 2. First-week treatment probability in CONV flocks over all years (7.2%, 95% CI [5.9, 8.7]) was higher than in MED flocks (2.0%, 95% CI [1.6, 2.5]) and SLOW flocks (1.3%, 95% CI [1.0, 1.7]) (*P* < 0.001). Treatment probability after the first week was similarly higher in CONV flocks (14.7%, 95% CI [12.1, 17.6]) than in MED flocks (3.2%, 95% CI [2.5, 4.0]) and SLOW flocks (2.2%, 95% CI [1.7, 2.9]) (*P* < 0.001). This difference was present in all years. Figure 3 shows the interaction between hatch year and production system. In addition, antibiotic treatment was associated with quarter, flock size and the number of houses on the farm (Table 2). Probability of antibiotic treatment in the first week was higher in quarter 1 than in quarter 3 and 4, and higher in quarter 2 than in quarter 3. Probability of antibiotic treatment after the first week was highest in quarter 1 and lowest quarter 3 and 4. An increase in flock size was associated with an increase in the odds of antibiotic treatment, both in the first week and after the first week. Flocks from farms with 3 houses had a lower probability of treatment in both periods than flocks from farms with 2 houses. Regional poultry farm density did not affect antibiotic treatment. The variances of the random effects indicate a substantial variability among farms as well as veterinary practices in terms of antibiotic treatments (Table 3).

**Table 2:** Odds ratios (OR) for variables associated with antibiotic treatment in the first week and after the first week. CONV: conventional; MED: medium-growing; SLOW: slow-growing.

		Antibiotics in first wk		Antibiotics after first wk	
Variable		OR (95% CI)	*P*	OR (95% CI)	*P*
Production system^1^	MED vs. CONV	**0.26 (0.22–0.31)**	**<0.001**	**0.12 (0.17–0.22)**	**<0.001**
	SLOW vs. CONV	**0.17 (0.13–0.22)**	**<0.001**	**0.13 (0.10–0.16)**	**<0.001**
	SLOW vs. MED	**0.66 (0.50–0.87)**	**0.001**	**0.67 (0.54–0.88)**	**0.001**
Year of hatch^1^	2014 vs. 2013	0.65 (0.35–1.20)	0.30	0.87(0.49–1.56)	0.96
	2015 vs. 2013	0.71 (0.42–1.18)	0.34	0.70 (0.40–1.21)	0.36
	2016 vs. 2013	0.74 (0.45–1.21)	0.45	0.91 (0.56–1.47)	0.98
	2017 vs. 2013	0.79 (0.49–1.28)	0.65	1.00 (0.62–1.58)	1.00
	2018 vs. 2013	0.62 (0.38–1.02)	0.07	1.19 (0.75–1.90)	0.83
	2019 vs. 2013	**0.38 (0.22–0.65)**	**<0.001**	0.91 (0.57–1.45)	0.97
	2020 vs. 2013	**0.48 (0.29–0.79)**	**<0.001**	0.77 (0.48–1.24)	0.56
	2021 vs. 2013	**0.55 (0.34–0.89)**	**0.007**	**0.59 (0.37–0.95)**	**0.020**
log_2_ (flock size)		**1.28 (1.22–1.34)**	**<0.001**	**1.48 (1.43–1.54)**	**<0.001**
Quarter of hatch	Q2 vs. Q1	0.96 (0.89–1.03)	0.47	**0.93 (0.88–0.99)**	**0.017**
	Q3 vs. Q1	**0.88 (0.82–0.96)**	**0.002**	**0.84 (0.79–0.89)**	**<0.001**
	Q3 vs. Q2	**0.92 (0.85–0.99)**	**0.031**	**0.90 (0.84–0.95)**	**<0.001**
	Q4 vs. Q1	**0.91 (0.84–0.98)**	**0.009**	**0.85 (0.80–0.91)**	**<0.001**
	Q4 vs. Q2	0.95 (0.88–1.02)	0.29	**0.91 (0.86–0.97)**	**0.001**
	Q4 vs. Q3	1.03 (0.95–1.11)	0.81	1.02 (0.96–1.09)	0.87
Number of houses	2 vs. 1	1.07 (0.85–1.34)	0.88	1.16 (0.95–1.41)	0.23
	3 vs. 1	0.86 (0.67–1.09)	0.38	0.96 (0.78–1.18)	0.96
	**3 vs. 2**	**0.80 (0.66–0.98)**	**0.018**	**0.83 (0.70–0.98)**	**0.019**
	>3 vs. 1	0.92 (0.72–1.17)	0.80	1.05 (0.85–1.29)	0.93
	>3 vs. 2	0.86 (0.70–1.05)	0.21	0.91 (0.77–1.07)	0.45
	>3 vs. 3	1.07 (0.89–1.29)	0.80	1.10 (0.94–1.28)	0.45

The meaning is that the bold numbers are significant (p < 0.05). The statistical significance is the p-value in column P.

1 Production type and hatch year are involved in an interaction (Figure 3).

**Table 3:** Random effect variances in the models for antibiotics in wk 1, antibiotics after wk 1, mortality, and footpad lesions (**FPL**).

	Variance			
Random effect	Antibiotics wk 1	Antibiotics After wk 1	Mortality	FPL
Farm	0.86	0.68	0.15	0.34
Veterinary practice	0.32	0.45	0.03	0.01
Slaughterhouse	–	–	–	0.01

### Mortality

**Figure 4:**
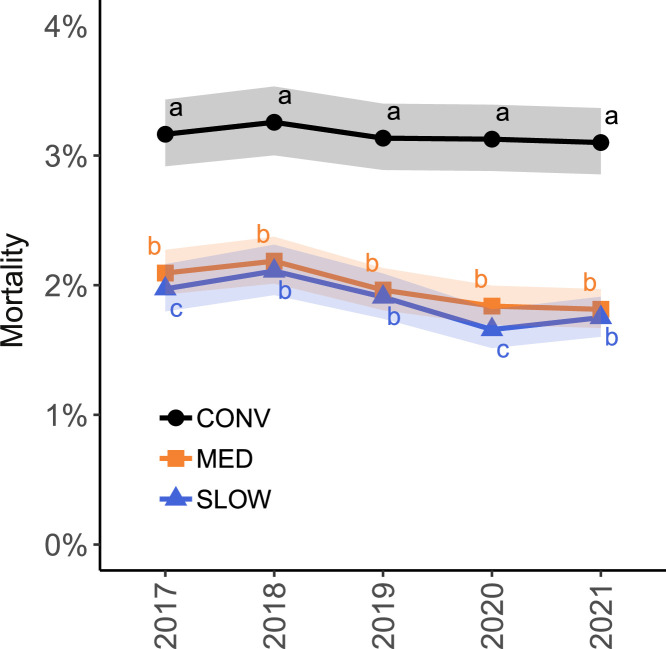
Marginal mean mortality per hatch year and production system based on the multivariate model corrected for quarter, number of houses and thinning, with random farm and veterinary practice effects. Colored bands indicate the 95% CI. Different letters (a, b, c) represent significant differences between production systems within each year. CONV: conventional; MED: medium-growing; SLOW: slow-growing.

The results of the multivariate analysis are summarized in Table 4. CONV flocks had a higher mean mortality than MED and SLOW flocks (CONV: 3.2%, 95% CI [3.0, 3.4]; MED: 2.0%, 95% CI [1.9, 2.1]; SLOW: 1.9%, 95% CI [1.8, 2.0]; *P* < 0.001). This difference was present in all years. The interaction between hatch year and production system is displayed in Figure 4. Mortality was also associated with quarter, number of houses and thinning (Table 4). The average mortality was highest in quarter 1 and 2, and lowest in quarter 3. Flocks from farms with 1 house had lower mortality than flocks from farms with more houses. Thinned flocks had a slightly lower mortality than flocks that were not thinned. Flock size and farm density did not affect mortality.

**Table 4:** Effect sizes for variables associated with mortality and footpad lesion scores. Results from the mortality model are presented as mortality ratios; results from the footpad lesion model are presented as score ratios. These ratios refer to the ratio of average mortality and FPL scores between levels of the explanatory variables, respectively. CONV: conventional; MED: medium-growing; SLOW: slow-growing.

			Mortality		Footpad lesions	
Variable			Mortality ratio (95% CI)	*P*	Score ratio (95% CI)	*P*
Production system^1^	MED vs. CONV SLOW vs. CONV SLOW vs. MED		**0.63 (0.61–0.64)** **0.59 (0.57–0.62)** **0.95 (0.92–0.98)**	**<0.001** **<0.001** **<0.001**	**0.46 (0.42–0.51)** **0.29 (0.25–0.33)** **0.62 (0.53–0.72)**	**<0.001** **<0.001** **<0.001**
Year^1^^,^^2^	2018 vs. 2017 2019 vs. 2017 2020 vs. 2017 2021 vs. 2017		**1.05 (1.03–1.07)** **0.97 (0.94–0.99)** **0.90 (0.88–0.92)** **0.91 (0.89–0.93)**	**<0.001** **<0.001** **<0.001** **<0.001**	**1.13 (1.07–1.21)** **1.85 (1.75–1.96)** **1.67 (1.57–1.76)** **1.24 (1.17–1.32)**	**<0.001** **<0.001** **<0.001** **<0.001**
Quarter^2^	Q2 vs. Q1 Q3 vs. Q1 Q3 vs. Q2 Q4 vs. Q1 Q4 vs. Q2 Q4 vs. Q3		1.01 (0.99–1.02) **0.94 (0.92–0.95)** **0.93 (0.92–0.94)** **0.95 (0.94–0.96)** **0.95 (0.93–0.96)** **1.02 (1.00–1.03)**	0.83 **<0.001** **<0.001** **<0.001** **<0.001** **0.027**	**0.66 (0.63–0.68)** **0.61 (0.59–0.63)** **0.93 (0.90–0.96)** **0.91 (0.88–0.93)** **1.39 (1.35–1.43)** **1.49 (1.45–1.53)**	**<0.001** **<0.001** **<0.001** **<0.001** **<0.001** **<0.001**
Number of houses	2 vs. 1 3 vs. 1 3 vs. 2 >3 vs. 1 >3 vs. 2 >3 vs. 3		**1.09 (1.02–1.16)** **1.10 (1.02–1.18)** 1.01 (0.95–1.07) **1.09 (1.01–1.18)** 1.01 (0.95–1.07) 1.00 (0.95–1.05)	**0.009** **0.008** 0.98 **0.012** 1.00 1.00	**0.78 (0.69–0.89)** 0.89 (0.78–1.03) **1.14 (1.02–1.28)** **0.77 (0.67–0.88)** 0.98 (0.88–1.10) **0.86 (0.78–0.95)**	**<0.001** 0.16 **0.012** **<0.001** 0.97 **<0.001**
Flock size (scaled^3^)		CONV MED SLOW	-	-	0.99 (0.97–1.00) **0.91 (0.88–0.94)** **0.89 (0.83–0.95)**	0.17 **<0.001** **0.004**
Thinning	Yes vs. no	CONV MED SLOW	**0.97 (0.95–0.98)**	**<0.001**	**1.39 (1.33–1.45)** **1.21 (1.04–1.41)** **0.69 (0.57–0.83)**	**<0.001** **0.017** **0.001**

The meaning is that the bold numbers are significant (p < 0.05). The statistical significance is the p-value in column P.

**Figure 5:**
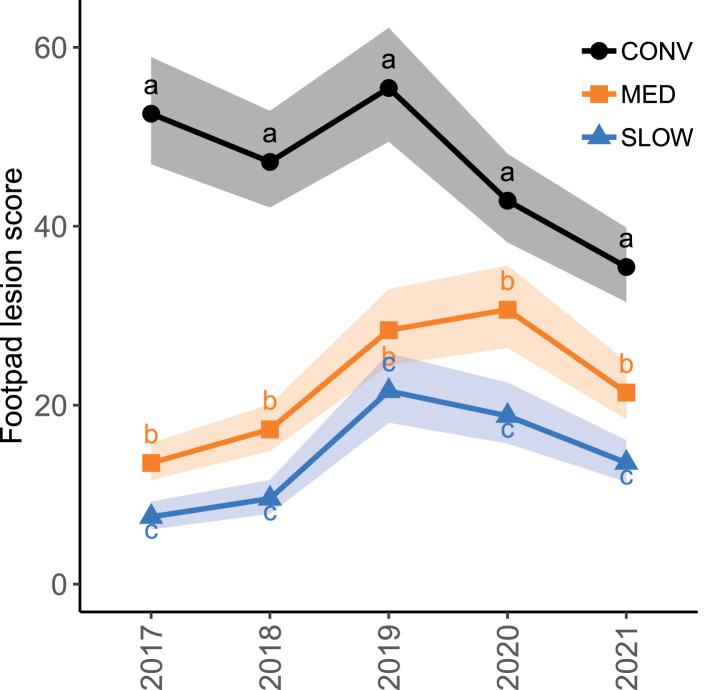
Marginal mean footpad lesion scores per hatch year and production system based on the multivariate model corrected for quarter, number of houses, flock size and thinning, with random farm, veterinary practice, and slaughterhouse effects. Colored bands indicate the 95% CI. Different letters (a, b, c) represent significant differences between production systems within each year. CONV: conventional; MED: medium-growing; SLOW: slow-growing.

1 Production system and hatch year are involved in an interaction (Figure 4, Figure 5).

2 In the mortality model, these refer to year and quarter of hatch. In the footpad lesion model, these refer to year and quarter of slaughter.

3 The score ratio indicates the change in FPL scores associated with an increase in flock size of 1 sd, which is equal to 11,843 birds.

### Footpad Lesions

The results of the multivariate analysis are summarized in Table 4. CONV flocks had the highest mean FPL scores, while SLOW flocks had the lowest scores (CONV: 46.1, 95% CI [42.1, 50.6]; MED: 21.3, 95% CI [18.9, 24.0]; SLOW: 13.2, 95% CI [11.5, 15.1]; *P* < 0.001). The interaction between hatch year and production system is displayed in Figure 5. Additionally, there was a very strong effect of quarter on FPL scores: in quarter 1, FPL scores were highest, followed by quarter 4, and the scores were lowest in quarter 3. FPL scores were also associated with number of houses on the farm, flock size, and thinning (Table 4). Flocks from farms with 2 or more than 3 houses had lower scores than flocks from farms with 1 or 3 houses. In MED and SLOW flocks, an increase in flock size was associated with a decrease in FPL scores, while there was no effect of flock size in CONV flocks. CONV and MED flocks that were thinned had higher FPL scores than flocks that were not thinned. In contrast, thinned SLOW flocks had lower FPL scores than flocks that were not thinned. Regional poultry farm density did not affect FPL scores. The results of the multivariate analysis with inclusion of 0 scores can be found in the supplementary materials (Supplementary Table S2). This analysis shows that including 0 scores did not change the conclusion that FPL scores are considerably lower in MED and SLOW flocks.

### Relationship Between Mortality, Antibiotic Treatment and Footpad Lesions

**Figure 6:**
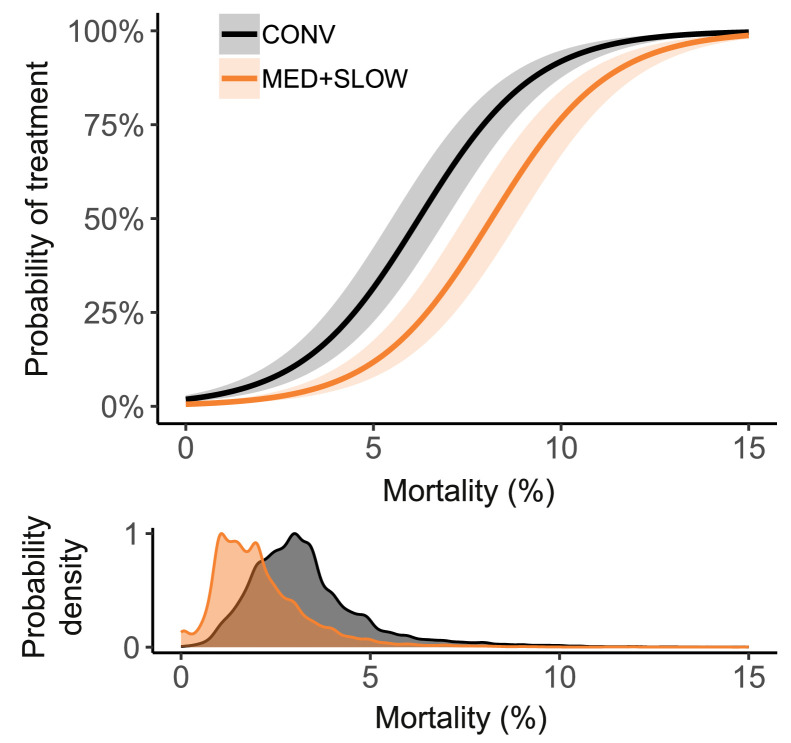
Top: marginal probability of antibiotic treatment plotted against mortality. Colored bands indicate the 95% confidence interval. Bottom: density plot representing the distribution of mortality rates of CONV and MED+SLOW flocks. CONV: conventional; MED: medium-growing; SLOW: slow-growing.

The odds of antibiotic treatment increased with increasing mortality (OR 1.89, 95% CI [1.86, 1.93], *P* < 0.001) (Figure 6). This was not different between conventional and slower-growing flocks (*P* = 0.47). The relationship between antibiotic treatment and FPL scores differed per production system (Supplementary Figure S3). CONV flocks treated with antibiotics had 16% lower FPL scores compared to nontreated CONV flocks (95% CI [0.82, 0.86], *P* < 0.001). MED flocks treated with antibiotics had 8.2% lower FPL scores compared to nontreated MED flocks (95% CI [0.87, 0.97], *P* = 0.004). In contrast, for SLOW flocks there was no difference between treated and nontreated flocks (ratio 1.08, 95% CI [0.94, 1.25], *P* = 0.26).

## DISCUSSION

This large-scale, multi-year study was performed to investigate differences in antibiotic treatments, mortality, and FPL scores between conventional flocks and slower-growing broiler flocks in alternative production systems. Broiler flocks were categorized into 3 production systems, CONV, MED, and SLOW flocks, based on breed and slaughter age. In addition to different growth rates, flocks from these groups differ in housing conditions, including stocking density. Compared to CONV flocks, both MED and SLOW flocks had fewer antibiotic treatments, lower mortality and lower FPL scores. Although this study focused on Dutch production systems, the findings are also relevant for other countries who are adopting or aiming to adopt production systems with higher welfare standards.

### Slower-Growing Flocks Receive Fewer Antibiotic Treatments

A general trend towards fewer antibiotic treatments in Dutch broiler flocks during the study period can be observed, to which the growing number of slower-growing flocks is a major contributing factor. The lower treatment probability of slower-growing flocks is likely a combined effect of growth rate and stocking density. However, other explanations, such as more effective vaccination programs in these flocks, cannot be excluded.

Experimental studies suggest that fast-growing broilers are more susceptible to intestinal disease, such as infections with *Salmonella* (van Hemert et al., 2006; Snyder et al., 2022) and *Eimeria* (Giles et al., 2019), and that high growth rate is a risk factor for necrotic enteritis (Dierick et al., 2019). Decreased gut health in fast-growing broilers could ultimately increase the need for antibiotic treatment. Likewise, stocking density was identified as a risk factor for necrotic enteritis (Tsiouris et al., 2015). High stocking density increases stress responses in broilers, which negatively impacts gut health. This effect of high stocking density on gut health is suggested to be caused by decreased air quality, together with higher competition for feed and water (Dai et al., 2022). Conversely, Tarakdjian et al. (2020) saw no difference in antibiotic use between commercial broiler farms with high or low stocking densities. However, farms with lower stocking densities were restricted by European legislation due inadequate air quality, introducing a potential confounding factor.

In all years combined, SLOW flocks had a lower probability of antibiotic treatment than MED flocks. However, when accounting for the interaction between production system and year, in most years the difference was not present. Although SLOW flocks have a lower growth rate than MED flocks, which is hypothesized to make them less susceptible to bacterial disease, they are also housed for a longer period, which could increase the risk of disease introduction. Moreover, antibiotic treatment probability in MED flocks was already relatively low in this study, which may limit the gains of stricter welfare requirements, such as in the SLOW group, on reducing antibiotic treatment even further.

Flock size is important to consider when comparing conventional and alternative production systems because conventional flocks are generally larger than alternative flocks. Our results show that an increase in flock size increases the probability of antibiotic treatment. A larger number of birds could increase the probability of disease introduction into a flock. Larger numbers of birds on a farm have previously been associated with a higher risk of necrotic enteritis (Hermans and Morgan, 2007) and early respiratory disease (Tablante et al., 1999). Moreover, De Wit et al. (2010) showed that flock size was correlated with a decrease in IgM response to infectious bronchitis virus vaccination. Another study found conflicting results regarding antimicrobial use: in 1 model, the number of broilers on European farms was negatively associated with antimicrobial use, while in a second model, farms categorized as low antimicrobial users had a lower median number of broilers than high users (Mallioris et al., 2023). Here the distinction between flock size and total number of birds on a farm might be important, with the latter also affected by the number of houses. In our study, we found no consistent effect of the number of houses on antibiotic treatment.

Although a decreasing trend in antibiotic treatments after the first week was seen in all types of flocks in the last years of the study, antibiotic treatments in the first week, which accounts for a considerate fraction of all antibiotic treatments, was more stable. Chick health in the first week seems to be largely determined by chick quality and conditions between hatch and placement on the farm (Bergoug et al., 2013; de Jong et al., 2019). To decrease the use of antibiotics even further, efforts should be directed towards chick health in the first days after hatch.

We assumed that the registration of antibiotic treatments, which has been used since 2011 by the SDa for monitoring and benchmarking of all broiler farms and veterinarians in the Netherlands (Bos et al., 2015), is complete and reliable. Veterinarians should register each prescription separately. The assumption was made that the antibiotics registered to a certain house on a supply date was given to the flock present in that house on that date. If multiple treatments were supplied at the same date but used at different dates, antibiotic treatments could have been underestimated.

### Mortality is Lower in Slower-Growing Flocks

Mortality can be used as a very general indicator of disease. Besides, it is often used as a general indicator of welfare in broilers (de Jong et al., 2022). The lower mortality of MED and SLOW flocks compared to CONV flocks is in accordance with de Jong et al. (2022), who found improved mortality scores in Dutch Retail broilers and the Better Life system compared to conventional broilers in 2017-2018. This effect is likely influenced by differences in growth rate. Studies comparing broilers with different growth rates under similar commercial housing conditions reported lower mortality in slower-growing broilers (Baxter et al., 2021; Forseth et al., 2024)*.* Lower mortality of slower-growing compared to fast-growing breeds under similar housing conditions is also observed in some experimental studies (Fanatico et al., 2008; Dixon, 2020; Abeyesinghe et al., 2021), although other studies found no difference (Weimer et al., 2020; Torrey et al., 2020; Abdourhamane and Petek, 2022). The absolute mortality in these experimental studies differ widely, potentially because of small sample sizes and differences in breeds, stocking densities, flock sizes, ambient temperatures, and slaughter ages. This makes studies under commercial conditions more suitable for comparing mortality of different production systems.

Growth rate can impact mortality in various ways. Slower-growing flocks, as previously noted, show lower susceptibility to certain infectious diseases. Additionally, fast-growing flocks are more prone to conditions such as ascites, as evidenced by carcass condemnations (Forseth et al., 2023), which can contribute to mortality. Fast-growing broilers also show impaired leg health (Rayner et al., 2020; Baxter et al., 2021; Santos et al., 2022). Lameness can be a reason for culling, which is included in mortality in our study. The effect of production system on culling rates could not be studied separately from other mortality in this study.

In contrast to growth rate, stocking density appears not to influence total mortality under both commercial (Dawkins et al., 2004; Bergeron et al., 2020) and experimental conditions (Shynkaruk et al., 2023). However, Shynkaruk et al. (2023) did observe a positive association between stocking density and mortality from infectious causes, which aligns with the previously described detrimental effects of high stocking density on gut health (Dai et al., 2022).

In the current study, the effect of production system on mortality was adjusted for the number of houses on the farm and thinning, as conventional flocks are typically from larger farms and are thinned more often than slower-growing flocks. Mortality was lower in single-house farms for unclear reasons. The lower mortality in thinned flocks is likely a result of the method used to calculate mortality, which uses the number of birds at the start of a round. Total mortality also provides no information on the distribution of the mortality over a production round. More research is needed to draw conclusions about the effect of thinning and the number of houses on mortality.

Only total mortality registered by the farmer was available for this study. This self-reporting could have created a bias if there was a difference in reporting between CONV, MED and SLOW flocks, which we assumed was not the case. Although an objective recording of daily mortality would be more accurate, this is not feasible for large-scale field studies. It would however be an improvement to use cumulative mortality, corrected for thinning. The analysis of weekly mortality, especially first-week mortality combined with first-week antibiotic treatments, would also be a valuable addition.

### Relationship Between Mortality and Antibiotic Treatment

The probability of antibiotic treatment increased with increasing mortality at a similar rate in CONV and ALT (MED + SLOW) flocks. Meanwhile, the overall probability of antibiotic treatment in slower-growing flocks was lower in ALT flocks than in CONV flocks, which implies that in case of equal mortality, MED and SLOW flocks were less likely to receive antibiotic treatment than CONV flocks. Farmers rearing slower-growing chickens might be less inclined to treat in the early stages of bacterial disease, trusting that the problem will subside without intervention. Alternatively, these farmers might be less motivated to treat due to imposed prohibitions of antibiotic use in certain farming concepts and potential penalties associated with the use of antibiotics. The extent to which these prohibitions are enforced and influence antibiotic treatments remains unknown. Still, it is crucial to note that, on average, mortality in slower-growing chickens was considerably lower than in conventional chickens. In addition, the cause of mortality is unknown and might be unrelated to bacterial disease. Lastly, mortality is assumed to drive antibiotic use; however, treatment of disease with antibiotics, especially in the early stages of disease, can also prevent an increase in mortality. This makes the interplay between mortality and antibiotic treatment difficult to assess.

### FPL Scores are Lower in Slower-Growing Flocks

A considerable number of studies demonstrated reduced FPL in slower-growing chickens (e.g., Williams et al., 2013; Rayner et al., 2020; Abeyesinghe et al., 2021; de Jong et al., 2022; Abdourhamane and Petek, 2022; van der Eijk et al., 2023). The present study provides a measure of the difference in FPL scores within the different Dutch production systems. SLOW flocks had the lowest FPL scores. However, the difference between conventional and slower-growing flocks had decreased since 2019. The national monitoring program may have stimulated farmers to improve housing conditions, ultimately reducing overall footpad lesion scores.

The lower FPL scores in MED and SLOW flocks can be a result of both lower growth rates (Williams et al., 2013; Rayner et al., 2020; van der Eijk et al., 2023) and lower stocking densities (van der Eijk et al., 2023). When kept at the same stocking density, fast-growing birds have poorer litter quality than slower-growing birds (van der Eijk et al., 2023), which is the main cause of FPL (Shepherd and Fairchild, 2010). Studies comparing broiler breeds with different growth rates at low densities and maintaining high litter quality throughout the study report no difference in FPL between breeds (Dixon, 2020; Baxter et al., 2021). This suggests that maintaining litter quality can mitigate FPL in flocks with fast-growing broilers. Likewise, stocking density is considered to influence the development of FPL mainly through its effect on litter condition (Shepherd and Fairchild, 2010). Some experimental studies report increased FPL with increased stocking density (van der Eijk et al., 2023; Shynkaruk et al., 2023; Zhou et al., 2024), while other studies with commercial housing conditions found no effect of stocking density on FPL (Dawkins et al., 2004; Bergeron et al., 2020). Dawkins et al. (2004) suggest that the house environment is more important than stocking density itself for bird welfare, including footpad health. It is possible that the effect of stocking density on litter quality disappears when house ventilation is adequately adapted.

FPL scores were also affected by quarter, thinning, flock size, and number of houses on the farm. Differences in ventilation rates are the probable cause of the pronounced seasonal variation in FPL scores, which is also observed in other studies (Meluzzi et al., 2008; Kyvsgaard et al., 2013). Higher stocking densities could explain the higher FPL scores in thinned CONV flocks compared to flocks that were not thinned. Less clear is the negative association between flock size and FPL, as well as the effect of the number of houses, with the highest FPL scores in farms with 1 house, followed by 3 houses. Future research should aim to clarify these found associations.

Recording FPL scores is mandatory for all flocks kept at stocking densities above 39 kg/m^2^. This means that farmers with MED and SLOW flocks are not required to score their flocks, which is reflected in the lower number of flocks with a score in the SLOW group. The voluntariness of the scoring could have introduced selection bias if flocks with severe FPL are not scored to avoid a penalty. However, when farms with incomplete FPL records were compared to farms with complete records, no difference in average FPL score was found. Another limitation of the FPL scores are 0 scores, which were considered errors. In MED and SLOW flocks, 0 scores could be unfairly excluded, which could have led to an underestimation of the difference with CONV flocks. However, the results from the model excluding 0 scores are comparable to the ones from the model including 0 scores (Supplementary Table S2).

### Relationship Between Antibiotic Treatment and Footpad Lesions

Antibiotic treatment was associated with lower FPL scores in CONV and MED flocks. De Jong et al. (2012) saw a similar association in Dutch conventional flocks in 2010 to 2011 across farms. However, within farms, untreated flocks had lower FPL scores. In a study by Knowles et al. (2008), flocks treated with antibiotics had better leg health. One could argue that antibiotics are used to treat one of the possible causes of wet litter: gastrointestinal disease. Unfortunately, no simple causal relationship can be identified. Data on the prevalence of gastrointestinal diseases and data on litter and air quality could help to better explain this relationship.

### Study Strengths and Limitations

This study provides an extensive analysis of the difference between conventional and slower-growing flocks within the Netherlands, using 9 yr of field data from a large part of the study population. The large sample size further provides great statistical power. We show the impact of the switch towards alternative production systems on broiler health and welfare. Unfortunately, we could not disentangle the effect of growth rate from the effect of other welfare requirements, most importantly the lower stocking density. Firstly, information on stocking density and other housing conditions were not available in this study. Secondly, even if available, growth rate and stocking density were too closely correlated within the Dutch broiler production for adequate analysis. This was also true for the effect of slaughter age, used to distinguish MED and SLOW flocks, which cannot be separated from growth rate and housing conditions. To answer these questions, controlled experiments should be performed.

In future studies, the health impact of alternative production systems could be explored in more detail by including information on the disease status of flocks. In the present study, mortality and antibiotic treatments were used as indicators of disease, which was possible due to the ban on preventive use of antibiotics (Speksnijder et al., 2015). The wide availability of this data made it possible to perform a nationwide analysis over 9 yr. However, these indirect indicators of disease were too limited to give a complete insight of the disease status of flocks. Knowledge on disease status would also help to elucidate the relationship between disease, mortality, and antibiotic treatments, and between disease, footpad lesions, and antibiotic treatments.

## CONCLUSION

The findings of this study show that switching from conventional production systems to alternative production systems with slower-growing broilers can have a positive effect on broiler health and antibiotic use.

## DISCLOSURES

The authors declare that they have no known competing financial interests or personal relationships that could have appeared to influence the work reported in this paper.

## Appendix Supplementary materials

Image, application 1

## Footnotes

Supplementary material associated with this article can be found in the online version at doi:10.1016/j.psj.2024.104197.
