# Supplementary material for: Broiler flocks in production systems with slower-growing breeds and reduced stocking density receive fewer antibiotic treatments and have lower mortality
Source: Poult Sci. 2024 Aug 8;103(11):104197. doi: 10.1016/j.psj.2024.104197 (PMC11395773; doi:10.1016/j.psj.2024.104197)
Supplement: Supplementary file 1 [file mmc1.docx]

**Supplementary tables and figures**

***Table S1.*** *Breed distribution of conventional (CONV), medium-growing (MED) and slow-growing (SLOW) flocks.*

| **CONV** | |
| --- | --- |
| *Breed* | *Percentage* |
| Ross | 88.6% |
| Cobb | 5.7% |
| Ross byproduct | 4.1% |
| Cobb byproduct | 1.0% |
| Diverse breeds (mix) | 0.3% |
| Hubbard | 0.2% |
|  |  |
| **MED** | |
| *Breed* | *Percentage* |
| Hubbard | 72.2% |
| Ross/Rowan Ranger | 24.2% |
| Ross | 3.2% |
| Hubbard byproduct | 0.2% |
|  |  |
| **SLOW** | |
| *Breed* | *Percentage* |
| Hubbard | 97.8% |
| Ross/Rowan Ranger | 2.1% |
| Ross | 0.5% |
| Hubbard byproduct | 0.1% |


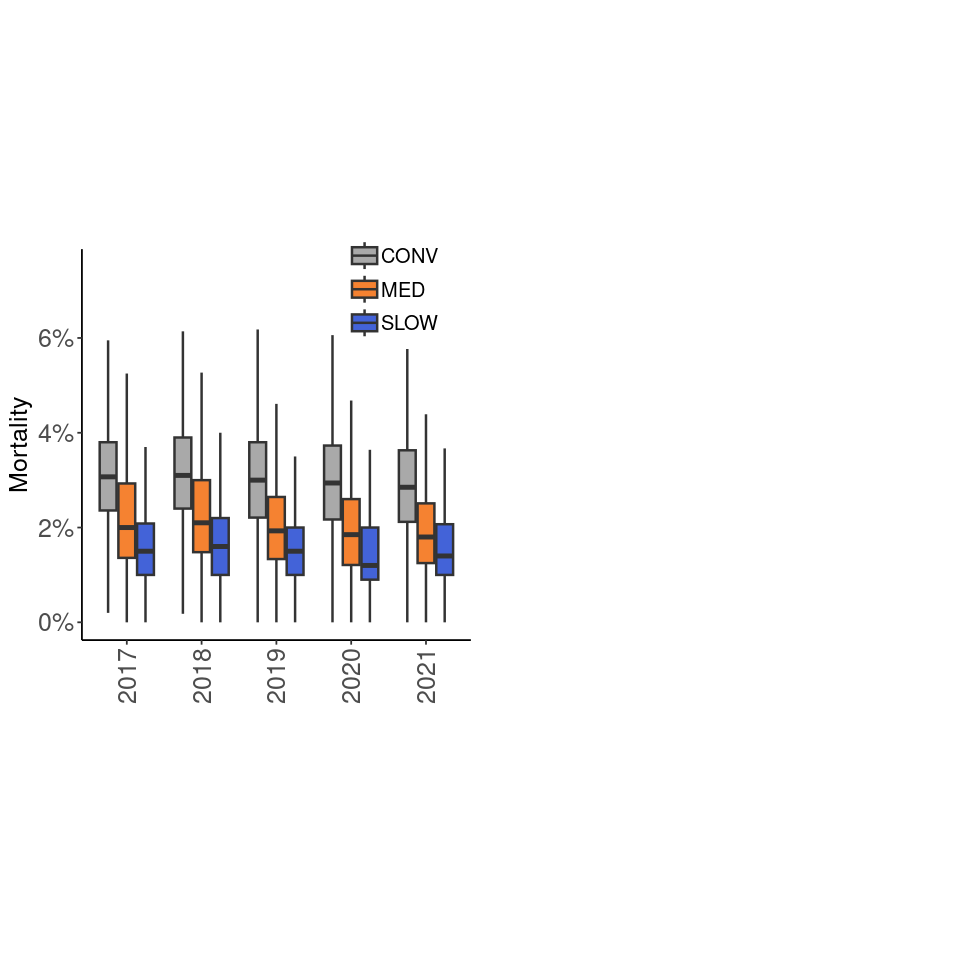


***Figure S1.*** *Median mortality rates per broiler type and hatch year.*

**B**

**A**

**A**

**
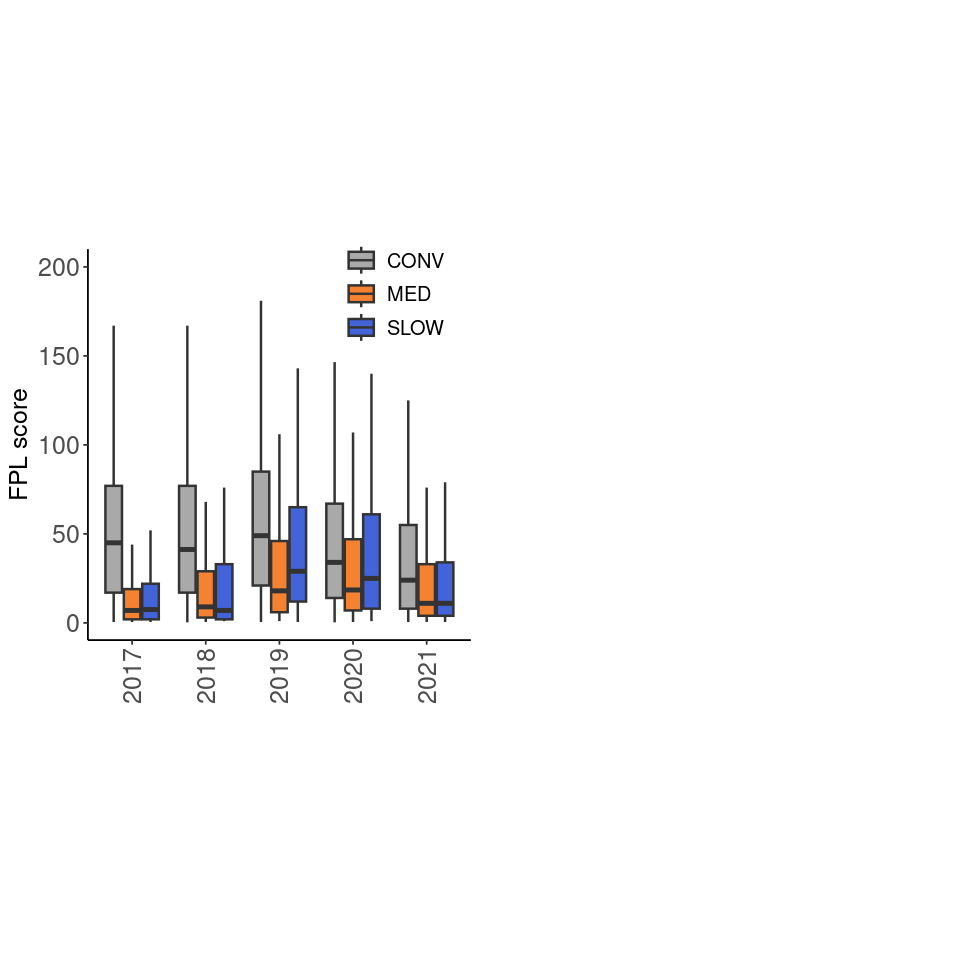

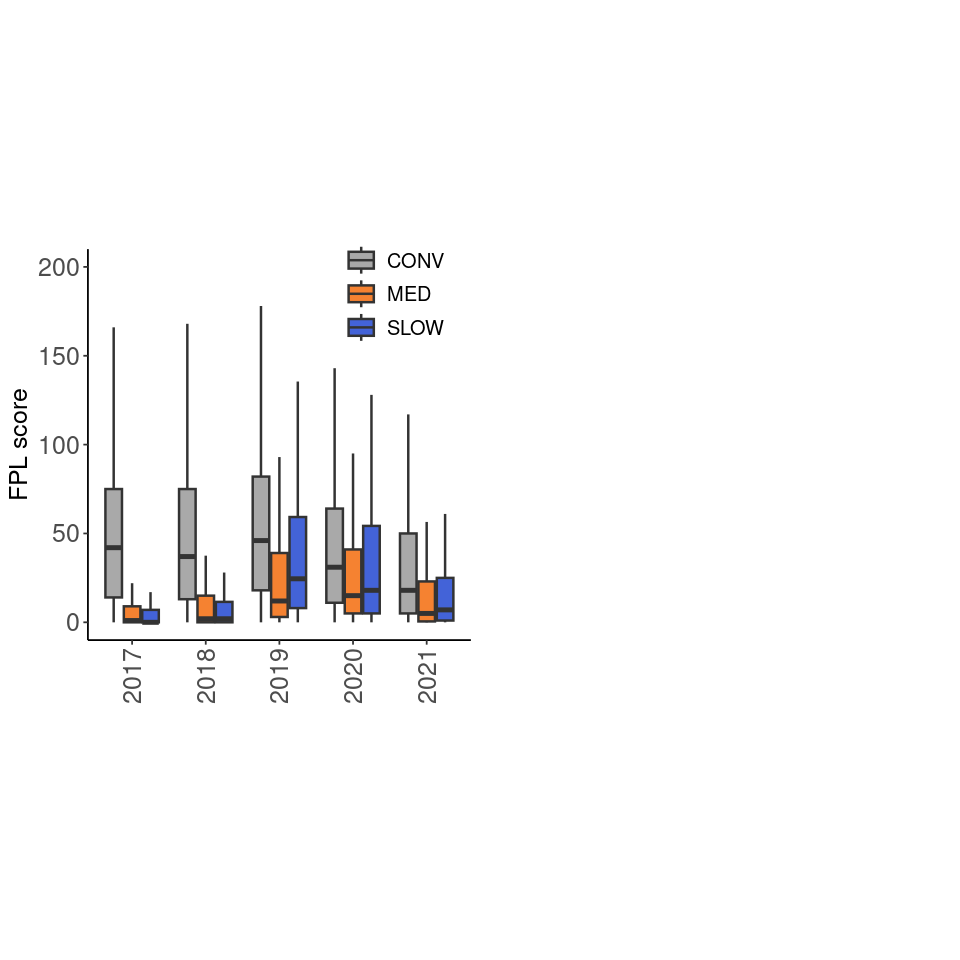
**

***Figure S2.*** *Median footpad lesion scores per broiler type and slaughter year. A) 0 scores are excluded; B) 0 scores are included.*

***Table S2.*** *Incidence rate ratios for variables associated with footpad lesion scores, with 0 score included in the model. Significant differences (p < 0.05) are indicated in bold.*

| Variable |  |  | Rate ratio (95% CI) | p |
| --- | --- | --- | --- | --- |
| Type | MED vs. CONV  SLOW vs. CONV  SLOW vs. MED |  | **0.37 (0.32-0.42)**  **0.20 (0.17-0.24)**  **0.55 (0.46-0.66)** | **<0.001**  **<0.001**  **<0.001** |
| Year of slaughter | 2018 vs. 2017  2019 vs. 2017  2020 vs. 2017  2021 vs. 2017 |  | **1.27 (1.19-1.35)**  **2.79 (2.62-2.97)**  **2.45 (2.30-2.60)**  **1.61 (1.52-1.71)** | **<0.001**  **<0.001**  **<0.001**  **<0.001** |
| Quarter of slaughter | Q2 vs. Q1  Q3 vs. Q1  Q3 vs. Q2  Q4 vs. Q1  Q4 vs. Q2  Q4 vs. Q3 |  | **0.59 (0.57-0.61)**  **0.53 (0.51-0.55)**  **0.90 (0.87-0.93)**  **0.90 (0.87-0.94)**  **1.53 (1.48-1.59)**  **1.70 (1.64-1.76)** | **<0.001**  **<0.001**  **<0.001**  **<0.001**  **<0.001**  **<0.001** |
| Number of houses | 2 vs. 1  3 vs. 1  3 vs. 2  >3 vs. 1  >3 vs. 2  >3 vs. 3 |  | **0.71 (0.61-0.84)**  **0.82 (0.69-0.97)**  **1.15 (1.00-1.31)**  **0.68 (0.57-0.81)**  0.96 (0.83-1.10)  **0.83 (0.74-0.94)** | **<0.001**  **0.012**  **0.042**  **<0.001**  0.84  **<0.001** |
| Flock size (scaled) |  | CONV  MED  SLOW | 1.00 (0.99-1.02)  **0.90 (0.87-0.94)**  **0.85 (0.77-0.92)** | 0.65  **<0.001**  **<0.001** |
| Thinning | Yes vs. no | CONV  MED  SLOW | **1.51 (1.43-1.60) 1.36 (1.12-1.64)**  **0.71 (0.57-0.88)** | **<0.001**  **0.002**  **0.002** |

**
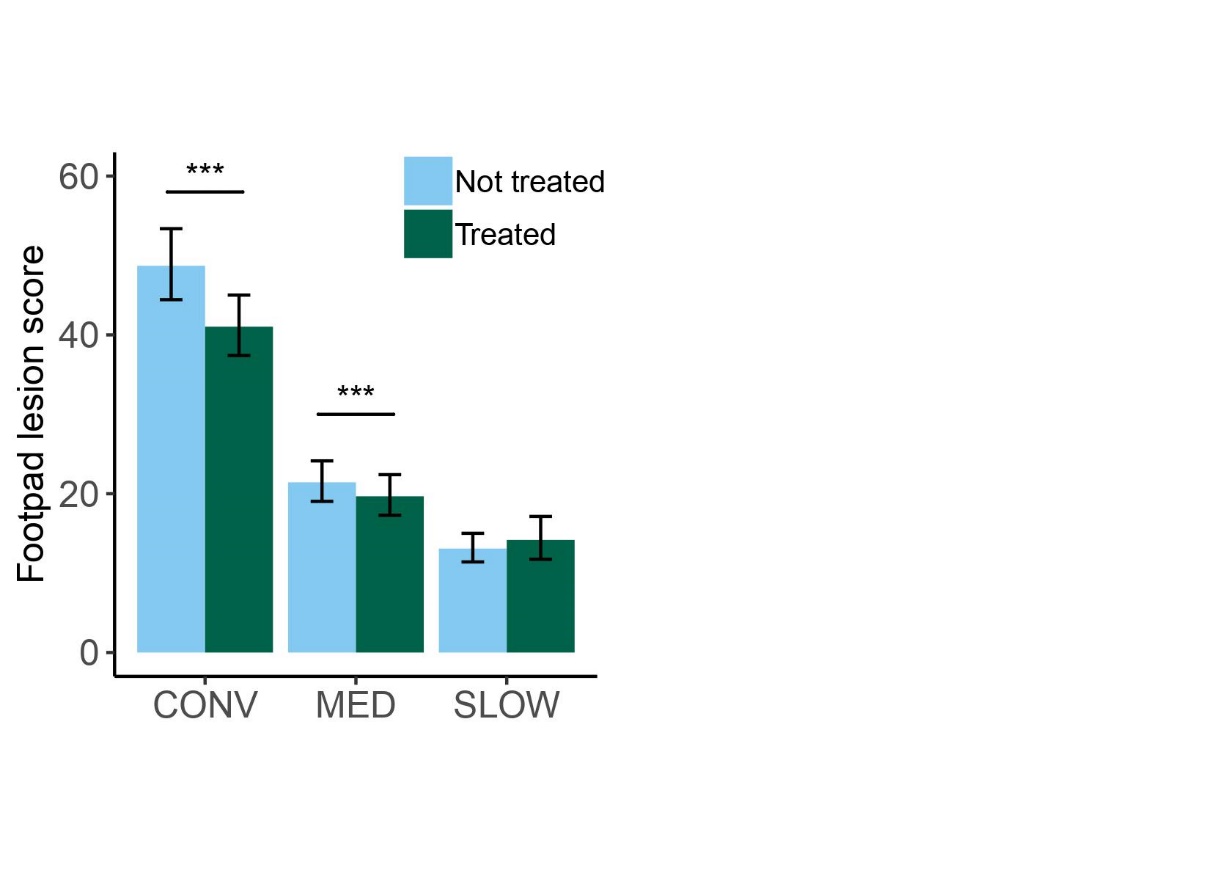
**

***Figure S3****. Marginal mean footpad lesion scores per production system and antibiotic treatment. Error bars indicate the 95% confidence interval. Asterisks indicate the significance level (*** p <0.001). CONV: conventional; MED: medium-growing; SLOW: slow-growing*
